# Investigation of interfacial strength in nacre-mimicking tungsten heavy alloys for nuclear fusion applications

**DOI:** 10.1038/s41598-022-26574-4

**Published:** 2023-01-11

**Authors:** J. V. Haag, J. Wang, K. Kruska, M. J. Olszta, C. H. Henager, D. J. Edwards, W. Setyawan, M. Murayama

**Affiliations:** 1 Department of Materials Science and Engineering, Virginia Tech, Blacksburg, VA USA; 2 Energy and Environmental Directorate, Pacific Northwest National Laboratory, Richland, WA USA

**Keywords:** Nuclear fusion and fission, Metals and alloys, Transmission electron microscopy, Magnetically confined plasmas

## Abstract

Tungsten heavy alloys have been proposed as plasma facing material components in nuclear fusion reactors and require experimental investigation in their confirmation. For this purpose, a 90W–7Ni–3Fe alloy has been selected and microstructurally manipulated to present a multiphase brick-and-mortar structure of W-phase ‘bricks’ surrounded by a ductile ‘mortar’. This work draws inspiration from nature to artificially imitate the extraordinary combination of strength and stiffness exhibited by mollusks and produce a nacre-mimicking metal matrix composite capable of withstanding the extremely hostile environment of the reactor interior and maintaining structural integrity. The underlying mechanisms behind this integrity have been probed through high-resolution structural and chemical characterization techniques and have revealed chemically diffuse phase boundaries exhibiting unexpected lattice coherency. These features have been attributed to an increase in the energy required for interfacial decohesion in these systems and the simultaneous expression of high strength and toughness in tungsten heavy alloys.

## Introduction

Immensely harsh environments necessitate extremely robust materials. Few case studies prove this statement better than materials for nuclear fusion reactors. Design constraints in the reactor interior, particularly the divertor region, include normal operational temperatures reaching 1300 °C^1^, repeated plasma strikes leading to enormous thermal shock^2,3^, and prolonged exposure to irradiation damage in the form of neutron bombardment and ion implantation at extreme energies and dose rates. These unfavorable conditions preclude the implementation of a majority of conventional materials. Materials selected for fusion reactor environments must not only survive this unique environment but thrive; providing long-term structural service in one of the most undeniably hostile environments yet conceived.

Thus far, a variety of materials have undergone trials to prove their viability as divertor tiles in fusion reactors but have met with limited success. Carbon-based tiles were initially selected due to their high melt temperature and widespread availability but were found to erode during operation. Additionally, these tiles were noted to bond with tritium, leading to unacceptably high levels of activity^4,5^. As a replacement, pure W tiles were chosen due to their high melt temperature and low sputter rate but were observed to develop cracks and fracture under repeated thermal loading^1,6–8^. This undesirable crack generation can be partially alleviated through manipulation of tile geometry and placement^7^, yet it is also prudent to select a material which maintains the benefits of W while also overcoming its inherently low fracture toughness. To combat the brittle behavior of tungsten while retaining the desired combination of a high service temperature and limited sputter rate, a class of alloys known as tungsten heavy alloys (WHAs) was proposed by Neu et al. for divertor tiles in 2016 experimental trials^1^. These alloys appear to be excellent candidates for plasma facing material components (PFMCs) as they retain a high tungsten content (≥ 90%) alongside a secondary phase, traditionally consisting of Ni and Fe or Cu. This secondary phase increases the fracture toughness of W through a phenomenon known as ductile phase toughening (DPT); essentially the purposeful introduction of a ductile material into a harder and more brittle material to improve ductility. In particular, the higher melt temperature of the Ni–Fe containing WHA over the Cu-based ductile phase has been pursued due to the high operational temperatures experienced in the reactor interior. Thus far, W–Ni-Fe WHAs have received positive results in their initial trials as PFMCs and in test reactors like the ASDEX Upgrade and external testing^1–3,7–9^. Although their proposed adoption is still in its infancy as much remains unknown about their behavior under extended service in the fusion reactor interior, particularly with regards to dissimilar phase boundary strength and irradiation behavior.

In the pursuit of understanding, improving, and implementing WHAs as PFMCs in fusion reactors, these materials have been the subject of ongoing microstructural design and optimization studies^10–14^, fundamental material modelling thrusts^15–17^, recent studies of ion irradiated WHAs^18^, and neutron activation during material service for the assessment of the allowable chemical compositions, especially in the case of Ni, for safe handling, disposal, and recycling^19^. The goal being the selection of an optimal microstructure and chemistry for PFMC service. To that end, a 90W–7Ni–3Fe (wt%) WHA which has been subjected to thermomechanical treatment mimicking the naturally occurring brick-and-mortar microstructures of nacre has been selected by Pacific Northwest National Laboratory (PNNL) as a prime candidate for PFMCs. This alloy was chosen as it has been proven to produce an optimal balance of strength and stiffness with surprising deformability^16,20^ while retaining the properties which make them promising candidates for adoption as PFMCs in nuclear fusion reactors.

**Figure 1:**
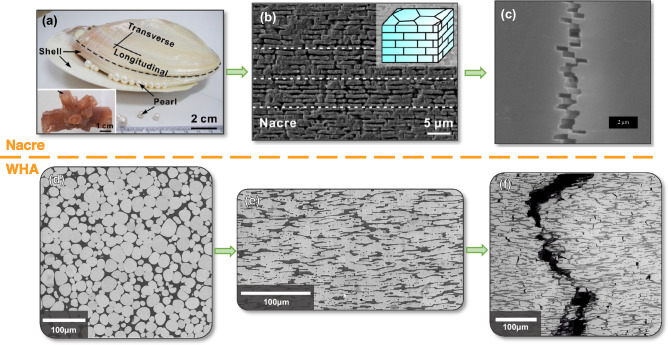
Comparison of naturally occurring nacre structures and their mechanical behavior (**a**–**c**)^22,23^ to that of the brick-and-mortar rolled WHA (**d**–**f**). Figures (**a**) and (**d**) are low magnification overviews of these materials in their naturally occurring and industrially produced states, respectively, while (**b**) and (**e**) are views of the brick-and-mortar structure in nacre and the WHA after thermomechanical processing, respectively. Figures (**c**) and (**f**) are views of these structures post uniaxial tensile testing to highlight their remarkably similar deformation behavior. Figures (**a**) and (**b**) are reprinted from Ref.^22^ with permission from Elsevier, and figure (**c**) has been reprinted from Ref.^23^ with permission from Elsevier.

Hierarchical nacre structures, shown in Fig. 1a–c, appear naturally in mollusk shells composed of aragonite (CaCO_3_) ‘bricks’ bound together by a soft biopolymer ‘mortar’^21–23^. These organically derived structures exhibit unique mechanical properties, which has driven in-depth research into the underlying mechanisms^20–26^. This work has now transitioned across disciplines to the field of nuclear materials, as these natural heterostructures can be effectively emulated in WHAs through hot-rolling of the material in its as-sintered state, Fig. 1d, to manipulate the isotropically distributed hard spherical W domains in a network of soft Ni–Fe-W ductile phase to resemble the series of stacked elliptical W domains held together by the ductile phase in the hot-rolled material, Fig. 1e. While the formation mechanisms are extremely different, the microstructures of these two materials are undeniably similar, Fig. 1b,e, as is their deformation behavior, Fig. 1c,f; thereby producing nacre-mimicking metal matrix composites composed of W ‘bricks’ bound by a ductile biopolymer ‘mortar’. The structural optimization of this tungsten composite is based on the calculations of an optimal brick aspect ratio of 5:1 for balanced strength and ductility determined by the microstructural modelling of Nguyen et al. in^16^. The applied thermomechanical processing conditions for their synthesis are outlined in^10^.

After an initial microstructural optimization study, these brick-and-mortar WHA alloys have been the subject of investigation into their mechanical behavior^11,12^ and microstructural formation^13,14^ during thermomechanical treatment. A suite of mechanical investigations from Alam et al. including tensile, microhardness, and fracture toughness testing have been conducted on 90W–7Ni–3Fe. These studies have experimentally demonstrated the utilization of the high stiffness of W and exemplary deformability of the ductile phase to achieve elongations at failure of 20% while retaining yield strengths in excess of 600 MPa^11,12^. In-situ tracking of specimen deformation has yielded evidence pointing to a high strength bond between the W and ductile phases, which is theorized to be directly responsible for the manifestation of DPT in WHA systems^13^. Yet the driving force behind this high interfacial strength remains unknown.

Consequently, this study has been designed to reveal the phenomena responsible for the bond strength of dissimilar multiphase material interfaces. This information is crucial to enable intelligent design of WHA microstructures for the effective retention of material integrity during service in fusion reactor systems. Examination of the interface characteristics is necessary at the atomic scale, both structurally and chemically. Therefore, a combined scanning transmission electron microscopy (STEM) and atom probe tomography (APT) approach has been implemented to explore the genesis of the high adhesion of interphase boundaries (IPB) in WHA systems.

## Results

In the analysis of interphase boundary strength, it is first necessary to describe the WHA system. The 90W–7Ni–3Fe alloy is a dual-phase metal matrix composite comprised of approximately 80 vol% of a nearly pure W-phase with a body-centered cubic (BCC) crystal structure and a face-centered cubic (FCC) Ni–Fe–W solid solution, here called the γ-phase. As stated earlier, the W-phase possesses a high hardness and melt temperature, but poor ductility; whereas the γ-phase matrix exhibits a comparatively low hardness and melt temperature, but high ductility. When utilized in tandem, this composite structure can express a balance of mechanical properties which could not otherwise be achieved, producing alloys containing 90% W or more with fracture strains in excess of 20%^11,12^. These mechanical properties have been further molded through microstructural manipulation to tailor the microstructure to a form which best fits its intended use as a PFMC, a central focus in prior study of these materials^10,13–16^. These prior analyses have revealed the introduction of a slight texturing in both the W and γ-phases and the presence of faceted planes at the IPB from the imposed thermomechanical processing of these alloy systems. It is theorized that the texturing of this material, though small in magnitude, leads to a predominance of planar matching between the BCC W-phase and FCC γ-phase and that boundary faceting stems from the reorganization of the IPB during the post-rolling anneal to lower the free energy of the boundary^14^. The bearing of these phenomena on the overall behavior is yet to be investigated, but it does prove the importance of the comparative crystallography in the consideration of IPB structure.

### STEM imaging

**Figure 2:**
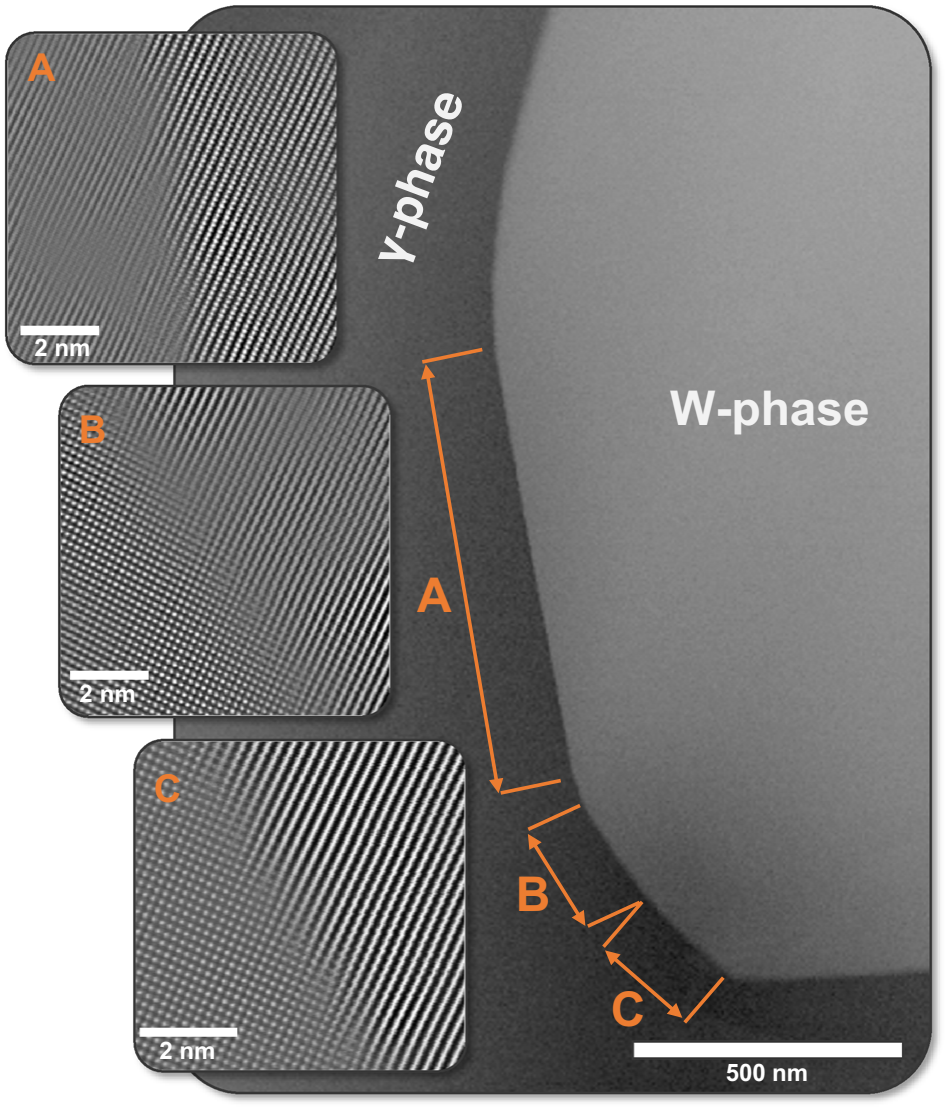
Atomic column micrograph of a multi-faceted region in the rolled WHA. The low magnification region presents facets A, B, and C with the insets to the left exhibiting FFT filtered atomic column micrographs of each facet at or near the on-edge condition. Raw data for each facet FFT micrograph has been provided in the Supplementary Material.

This faceting behavior at the IPB can be seen more clearly in the low magnification high angle annular dark field (HAADF) STEM micrograph shown in Fig. 2. This boundary presents three distinct facets between the two grains of interest, here labelled A, B, and C. Each facet has been oriented to view the atomic structure of the IPB plane as well as for the analysis of lattice coherency. That is, each separate inset is collected at slightly altered tilt conditions to accommodate localized crystallographic misorientation. The insets provided in Fig. 2 show the structure of these boundary planes with corresponding atomic column images.

**Figure 3:**
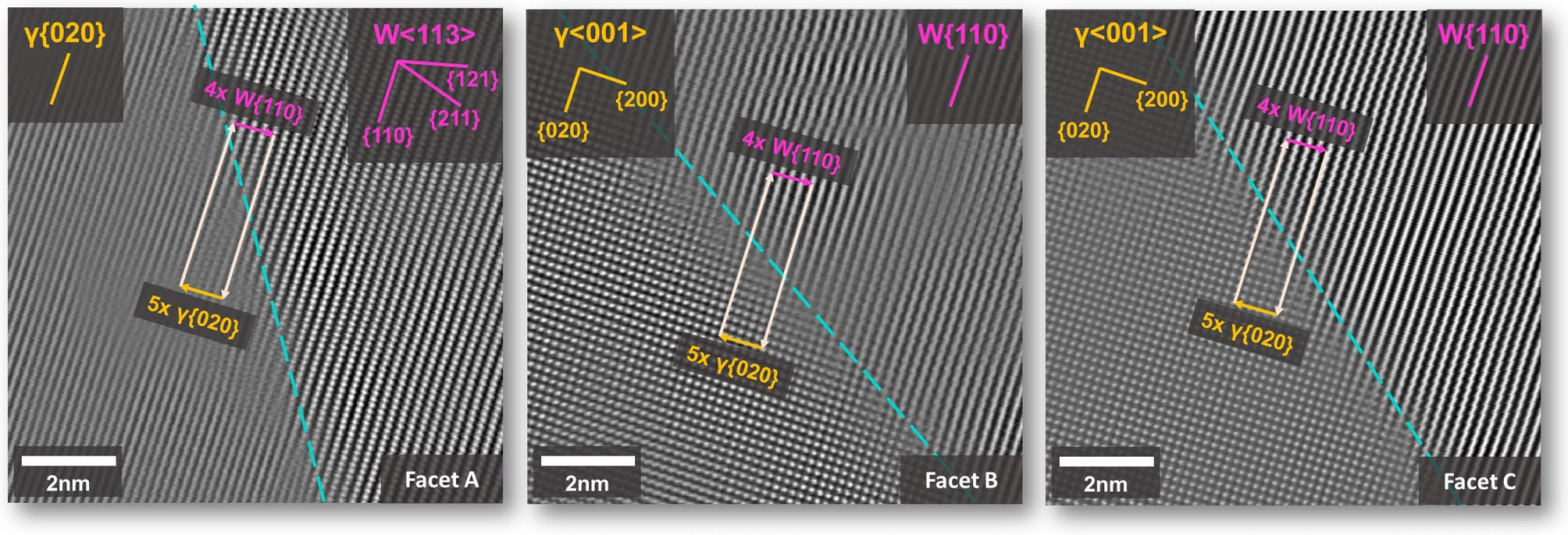
Atomic column images of facets A, B, and C shown in Fig. 2. Each region has been mapped, providing crystallographic planes and directions in the BCC W and FCC γ-phase regions on either side of the IPB. It is evident that for each facet the W{110} is parallel to the γ{200}. For each boundary, a Burgers circuit has been drawn highlighting that regardless of the physical boundary plane, the lattice matching remains constant.

A more in-depth analysis of the Fourier filtered atomic column micrographs, Fig. 3, reveals the degree of lattice coherency between the W and γ-phases (raw data provided in the Supplementary Material). Both grains were crystallographically mapped to determine the mutual lattice planes between W and γ, revealing that the W{110} lies parallel to the γ{020}. It should be noted that even though the IPB normal plane may change between facets A, B, and C, this orientation relationship (OR) stays consistent for each facet. This is the case for each micrograph displayed in Fig. 3, with all boundaries maintaining the W{110} // γ{020}. It is also apparent that each facet possesses a unique IPB plane orientation, as indicated in the low-magnification micrograph in Fig. 2*.* In Fig. 3, for each IPB plane, a blue dotted line has been placed over the boundary for ease of interpretation. For facet A, the edge-on condition was satisfied when viewing down the W < 113 > zone axis. A Burgers circuit can then be drawn at the interface showing a long-range repeating matchup between 4 × W{110} and 5 × γ{020}. The periodic appearance of an additional half-plane and evidence of misfit strain on the γ-phase side of the interface indicates a semi-coherent structure at this boundary facet. This strain only appears on the γ-phase side of the IPB, with no discernable lattice strain in the W approaching the boundary. While the IPB planes change for facets B and C, an identical Burgers circuit can be applied, and the same lattice matching relationship and evident γ-phase strain hold true despite the altered specimen orientations to maintain the edge-on condition. This result indicates that the W-γ boundary remains semi-coherent regardless of the IPB facet orientation and points to the prevailing importance of the OR between grains in the consideration of dissimilar material boundaries.

**Figure 4:**
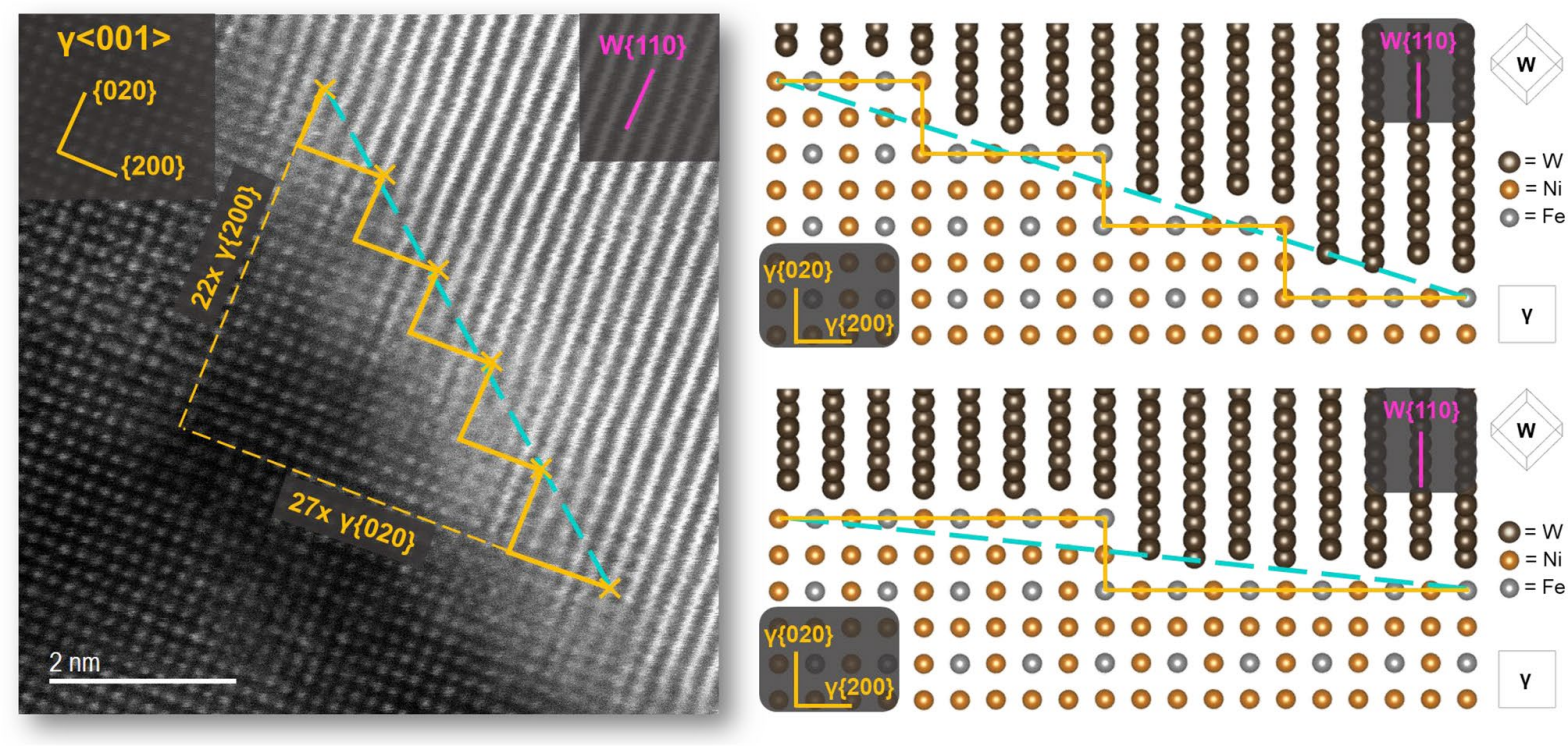
(Left) Atomic column STEM micrograph of on-edge interphase boundary plane highlighting stair-stepped appearance of boundary plane. (Right top and bottom) Diagrams of atomic structures comparable to that of the micrograph shown on the left defining two different interphase boundary planes which maintain the same OR and misfit dislocation spacing with different boundary planes.

Additionally, the importance of the physical boundary plane (the normal plane) cannot be discounted in the analysis of these systems. In the indexing of the HAADF micrograph provided of Facet C in Fig. 4, it is apparent that the IPB plane on the γ-phase side corresponds to a high index lattice plane, the (22 27 0), but is composed of individual 90° stair-stepped facets with of {020} and {200} faces. In this way, what would at lower magnifications appear to be an irrational lattice plane is in reality a repeating series of steps composed of structural ledges possessing low-index planes. This type of IPB structure is expected to correspond to a lower interfacial energy for the boundary than would be possible in a higher index conformation. A reduction in the free energy of the system may then be attained through the establishment of structural ledges to span regions which may otherwise be structurally incoherent.

A corresponding model of the same crystal OR with two different boundary conformations was constructed using VESTA^27^. The structure file for room temperature W was used for the W-phase (a = 3.1648 Å) ^28^; and while no appropriate structure file could be found for the γ-phase, the file used by Jiang et al. in^18^ for the indexing of the γ-phase in EBSD was modified to adjust the lattice parameter to that experimentally determined by Muddle and Edmonds (a = 3.595 Å)^29^. Using these lattice parameters, there is an approximate 20% lattice mismatch between W{110} and γ{020} spacings. As expected, this confirms that every 4 × W{110} equates to 5 × γ{200} within 0.4%. This VESTA model possesses the added benefit of allowing the manipulation of the IPB plane to exhibit the behavior of structural ledges generating a boundary with an irrational plane depending on the width of the ledges.

#### STEM-EDS

**Figure 5:**
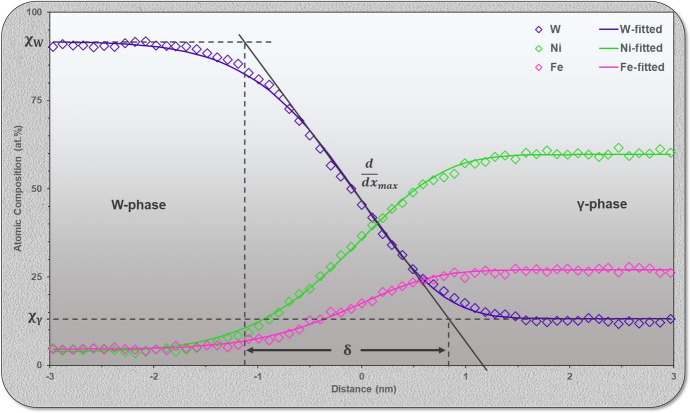
Graphical representation the of gradual chemical transition from W-phase to γ-phase proceeding across the IPB (left to right) from STEM-EDS. W is shown in purple, Ni in green, and Fe in pink. The atomic composition across the IPB has been calculated from the application of a sigmoidal fitting function, providing the bulk concentration of each elemental species in the W and γ-phases (χ_W_ and χ_γ_ respectively), also leading to the determination of IPB width, δ. This has been shown for only the element W in this figure.

In addition to structural analyses of the IPB, it is possible to collect local elemental distribution information through STEM energy dispersive x-ray spectroscopy (EDS) mapping. This is particularly useful in the study of these materials as there exists very little literature discussing the local composition of the W-γ interface and its subsequent bearing upon material behavior. Figure 5 presents a representative EDS line profile of an edge-on IPB region for the observation of incremental composition in the vicinity of the boundary plane. A total of four IPB regions were measured, with the associated elemental compositions of the bulk phases (χ_W_ and χ_γ_) and interfacial width, δ, values included in Table 1. The values displayed in Table 1 have been determined from a functional fitting of the acquired spectra across the boundary with further details on their calculation displayed in the Supplementary Material alongside a composite graph of all STEM-EDS composition profiles and mapping information. The calculation of these values is based on the method applied by Ardell in^30^.

**Table 1:** List of calculated bulk compositions and interfacial width as determined from STEM-EDS and APT analyses.

	*χ*_γ-phase_ (at.%)			*χ*_W-phase_ (at.%)			*δ* (nm)		
	W	Ni	Fe	W	Ni	Fe	W	Ni	Fe
**STEM-EDS**									
1	7.8	67.0	26.0	96.9	0.0	1.8	1.8	2.1	1.8
2	13.3	59.7	27.0	91.6	3.9	4.6	1.8	1.9	1.7
3	12.3	60.6	27.2	93.6	2.5	3.8	2.8	2.8	2.9
4	11.8	60.7	27.5	96.6	0.0	2.0	2.5	2.5	2.7
**Avg.**	**11.3**	**62.0**	**26.9**	**94.6**	**1.6**	**3.0**	**2.2**	**2.3**	**2.3**
**APT**									
1	0.0	65.3	30.9	90.9	4.4	2.7	1.2	1.2	1.2
2	4.3	62.9	31.1	80.3	8.9	5.3	1.5	1.3	1.6
3	6.5	64.7	30.0	88.1	0.0	3.1	1.6	1.6	1.3
4	0.0	66.0	28.9	93.5	6.6	0.0	1.6	1.6	1.4
**Avg.**	**2.7**	**64.7**	**30.2**	**88.2**	**5.0**	**2.8**	**1.5**	**1.4**	**1.4**

The line profile in Fig. 5 has been normalized to present the W-phase on the left and transition across to the γ-phase on the right. It is apparent from these calculations that there exists a gradual chemical transition from W-phase to γ-phase taking place over approximately 2 nm. Even when acquired at different phase boundary planes and regions representing different crystallographic ORs, the width of this diffuse boundary region appears to be constant. This indicates that the effects of boundary structure and OR between grains appear to possess a minimal effect upon the chemical environment at the boundary. If boundary structure then mediates boundary width, it appears to do so at a magnitude below the level of detection easily discernable by probe-corrected STEM-EDS.

It should be addressed that there exists a concern this chemical transition region may be a result of the spread of the STEM probe through the sample thickness, especially due to the extremely small width of the measured compositional gradient. This issue is further exacerbated by the presence of heavy scattering elements like W, causing a greater degree of beam broadening than its lighter atomic number counterparts. It is the assertion of the authors that the diffuse boundary region is not an artifact of beam spread but is instead a result of the boundary approaching chemical equilibrium through the formation of a compositional gradient much like that described by the Cahn–Hilliard equation in the determination of phase equilibria in multi-component systems^31,32^. This does remain a concern in the reliability of the measured gradient width from EDS results and therefore necessitates the application of complementary techniques in the analysis of the chemical environment at the boundary.

#### APT

**Figure 6:**
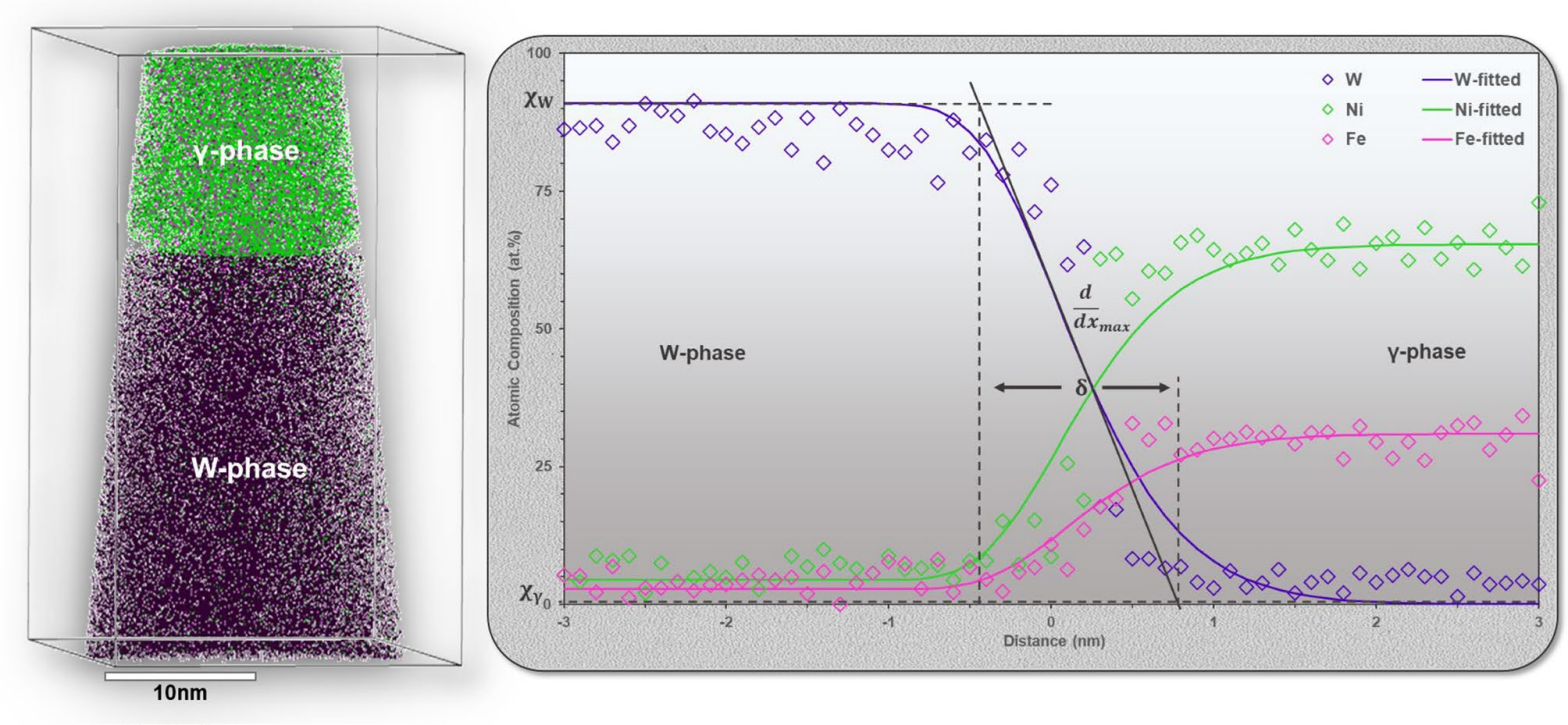
(Left) APT reconstruction of IPB region showing W in purple, Ni in green, and Fe in pink. (right) Compositional profile across a region of the reconstructed APT tip. This data was fitted in the same method as Fig. 5. Profile proceeds from the W-phase on the left to the γ-phase on the right with the δ-value determined from the fitted curve for the W-phase. The acquired compositions and IPB width values for Ni and Fe from these spectra and for three additional APT reconstructions can be found in Table 1, with a composite of all four acquired line spectra included in the Supplementary Material.

In the application of complementary characterization techniques in the analysis of composition across the IPB, atom probe tomography has been applied. This is a method by which the three-dimensional spatial distribution of elements can be probed with extreme chemical and spatial sensitivity. In contrast to STEM-EDS mapping, APT is intrinsically 3D in nature and can account for effects of boundary curvature through the application of isoconcentration surfaces but remains less sensitive to structure than STEM. A representative example of the reconstructed interfacial region from APT with corresponding spectra acquired normal to the IPB has been provided in Fig. 6. Four total composition profiles have been extracted across the IPB, two each from two different APT tips. All corresponding ion maps for both tips have been provided in the Supplementary Material. The average interfacial width of the boundary appears to be approximately 1.5 nm and is noted to be consistent between all four extracted compositional profiles shown in Table 1. Additionally, this interfacial width remains extremely close to that measured by STEM-EDS, lending further evidence to the presence of a chemically diffusive boundary with a nanometer-scale width.

Just as in STEM-EDS, APT possesses limitations in the analysis of dissimilar material interfaces that bears discussion. The most prominent issue in heterogeneous boundary characterization is trajectory aberration^33^. Due to the difference in evaporation fields between elemental species, the ion evaporation trajectory among species can be affected potentially giving rise to artifacts where ions are wrongfully placed over the phase boundary in the reconstructions. Such artifacts could occur at the scale of a few nanometers in width, which could potentially coincide with the interfacial range explored here. The magnitude of the diffusion boundary width can also be greatly affected by the reconstruction parameters. By varying the reconstruction parameters to extremes, the interfacial width can range upwards of 5 nm in size. The width values obtained from the reconstructions presented here have therefore been conservatively measured given the best estimations of needle geometry. Despite the limitations of STEM-EDS and APT as methods for the probing of nanometer-scale structure and chemistry at heterogenous boundaries, both techniques strongly suggest the presence of a chemically diffusive boundary on the scale of δ < 5 nm.

## Discussion

**Figure 7:**
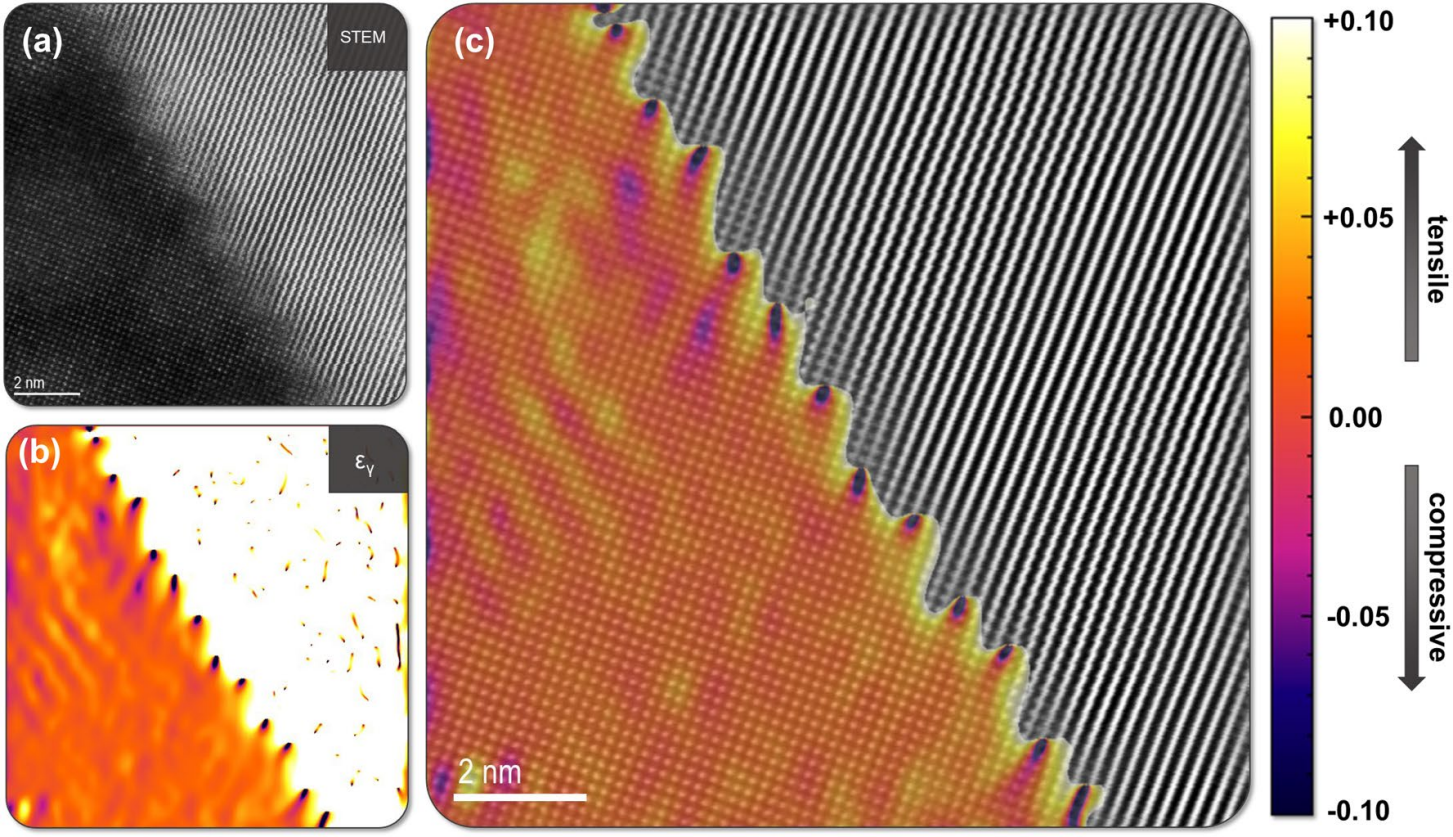
(**a**) Atomic column STEM micrograph of facet C in same orientation as shown in Fig. 4, (**b**) strain map of the lattice expansion along the γ{020} planes in (**a**), (**c**) overlay of γ-phase strain map from (**b**) and Fourier filtered micrograph from (**a**) at the interphase boundary highlighting the periodic strain field corresponding to misfit dislocations and coherency strain across the boundary plane. Note that the generated Strain++^35^ map has been optimized to only display variations in the γ-lattice therefore the overlay does not include contributions from the W-phase.

The interphase boundary is critical in the effective manifestation of ductile phase toughening behavior in tungsten heavy alloy systems. To determine the phenomena responsible for the strength of these boundaries and subsequent DPT effect these structures have been subjected to rigorous examination at the nanoscale, both from a structural and chemical perspective. Analyses of the atomic structure in these regions, Figs. 2 and 3, have provided experimental evidence of lattice coherency at IPB facets, with W and γ-phase lattices manifesting a semi-coherent boundary structure. It is noted that there is little to no discernable lattice strain in the W-phase with the γ-phase lattice accommodating the misfit due to the large disparity in elastic constants between the phases. This strain in the γ-phase can also be calculated through the implementation of geometric phase analysis^34^ using Strain++^35^ and utilized to visually display the strain field across the boundary plane, Fig. 7. The periodic variations in the calculated strain field at the boundary directly correspond to the location of misfit dislocations in the γ-phase and become clear in the overlay of Fig. 7c. Additionally, as noted in Figs. 3 and 4, this relationship between lattices with the γ-phase accommodating for lattice misfit is shown to hold true over multiple IPB plane orientations between two grains maintaining a consistent OR, with mutual lattice planes in BCC W and FCC γ-phase corresponding to observed textural components noted in prior analyses and similar composition systems^14,36^. This relationship holds true for various orientations of the physical IPB plane, exhibiting an OR composed of parallel sets of planes in the lattice, W{110} and γ{020}. Semi-coherency is associated with a lower overall free energy of the IPB compared to an incoherent boundary conformation^37^, and likely results from the imposed thermomechanical processing conditions leading to the formation of preferential orientations in both the W and γ-phases, which are then able to relax and reorient during post-rolling annealing steps, forming a semi-coherent boundary.

Higher magnification observation of these structures, Fig. 4, supports this idea of boundary migration; showing facets which appear to possess irrational high-index lattice planes at the IPB but are composed of stair-stepped structural ledges. The ledges are composed of rational low-index steps corresponding to the {020} and {200} planes in the γ-phase. Structural ledges have long been discussed as methods by which materials with dissimilar lattice spacings can accommodate lattice misfit, and this appears to hold true for the WHA system^38–40^. Observation of the ledges in Fig. 4 suggest they are in fact bounded at either end by dislocations; essentially producing ledges the width of the spacing between γ-phase misfit dislocations, a phenomenon discussed in BCC/FCC interfaces since the early 1970s to allow partial regions of coherency^38^. This same phenomenon also apparent in the shape of the calculated strain field along the boundary in Fig. 7. These IPB regions, composed of ledge-like facets, are noted to be comparatively massive in total boundary area compared to the nanometer-sized precipitates discussed by Hall and Zhang^38,40^; therefore, it is asserted the W domain interfaces with the γ-phase do not adopt a Wulff shape as described in^40,41^, but rather the added constraints of massive interfacial area and potential interaction with surrounding domains leads to a non-equilibrium shape. These faceted boundaries in thermomechanically processed WHAs may then allow a reduction of total free energy in the system, achieving a local energy minimum without reaching the lowest possible energy state and boundary conformation. This behavior agrees with the study of energy minimization in multiphase systems through faceting conducted by Howe et al.^42–45^.

In the evaluation of composition at the IPB, both APT and STEM-EDS agree that there is a gradual chemical transition from W to γ-phase. This diffuse boundary region is measured to be between 1.2 and 2.9 nm in width and is theorized to be a result of the boundary approaching a state of chemical equilibrium. While the exact width of this boundary is unknown, its existence is corroborated by Monte Carlo simulation of free energy minimization in multicomponent systems^30^ and has been discussed experimentally in the Au-Cu^45^, Co-Al-W^46,47^, and Ni-based alloys^48,49^. In following the classical Cahn–Hilliard approach for the equilibrium composition across the boundary in a two-phase system, a chemically diffuse boundary is energetically favorable, with steep chemical gradients, or narrow interface widths, imposing an energy penalty in the system^30,31,44^. The mathematical approach of Cahn–Hilliard yields a sigmoidal compositional profile across the boundary, the same shape as those experimentally measured from APT and EDS in this case mediated by the small solubility of Ni and Fe into W. This chemically diffuse IPB region is theorized to act as a narrow diffusion couple between the W and γ-phases across the material, intimately bonding the dissimilar phases and thereby increasing the fracture energy required for the boundary.

It is therefore posited that the IPBs in this inorganic W-based nacre structure derive their strength from the combined effects of lattice coherency and the presence of a chemically diffuse interphase boundary region raising the energy required for the decohesion of the W–γ interface. This assertion appears to agree with density functional theory (DFT) calculations of the W–Ni interface in^17^, showing fracture within the Ni domain, even when there is no consideration of a chemical gradient between the phases. While there exists no exact translation between the free energy of a boundary and its strength, it is hypothesized that the lower the free energy of the region, the greater the magnitude of force needed to fracture the interface. The presence of semi-coherent boundaries and indications of chemically diffuse regions are both associated with lower interfacial and systematic free energy values than their incoherent and steep chemical gradient counterparts. Ultimately, the exact correlation between boundary energy and strength is unknown, but the combined factors of boundary conformations which present structural semi-coherency and the presence of a ubiquitous small-scale diffusion bond between W and γ grains clearly mediates the energy requirement for fracture of the interphase boundary.

## Methods

### Materials

The alloy of interest in this study is a tungsten heavy alloy (WHA) produced by Pacific Northwest National Laboratory from stock Mi-Tech W, Ni, and Fe powders without use of binders. Powder compact specimens nominally composed of 90% W–7% Ni–3% Fe by weight were heated to 1500 °C in hydrogen to liquid phase sinter the material at a point in which Ni and Fe melt and W does not; then specimens were degassed at 1000 °C in vacuum. Resulting specimens were hot-rolled through sequential rolling and annealing steps to an overall 87% thickness reduction with a final anneal in Ar-H_2_, then a one-hour degassing step at 900 °C in vacuum. Specimens for STEM analyses were cut from the rolled billet such that the rolling direction would be parallel to the thickness dimension of the electron transparent specimen foils. Specimens for APT analyses were lifted out from the surface of a polished specimen and thinned as to present a IPB at the tip of the needle.

#### STEM/EDS

For the investigation of the phase boundary plane from an atomistic and structural perspective; as well as the collection of elemental maps for specimen chemistry analysis, STEM was the primary technique selected in the examination of these materials. Specimens for STEM observation were prepared through grinding and polishing of sections from the rolled billet to approximately 100 μm in thickness, dimpling with colloidal diamond paste on a Gatan model 656 dimple grinder until just prior to perforation, then Ar^+^ ion milled using a Gatan PIPS II at cryogenic temperatures down to a final thinning step at ≤ 250 eV to minimize surface damage in the foils. STEM analyses were conducted on a probe corrected JEOL GrandARM 300F equipped with a cold field emission gun (FEG) and operated at 300 kV as well as a probe corrected JEOL ARM 200F equipped with a cold FEG and operated at 200 kV. All images displayed here were collected using dark field conditions, with STEM-EDS maps acquired using JEOL model Centurio detectors. STEM analysis was performed using Gatan GMS3 and EDS data was processed using Pathfinder version 1.4. Raw data for imaging and EDS can be found in the Supplementary Material. Prior to atomic column analyses, regions were crystallographically mapped by orienting each grain of interest in a dual-axis tilt TEM holder to at minimum three non-coplanar zone axes for orientation determination. This was done to derive the orientation relationship between the grains as well as to determine the most advantageous imaging conditions for specimen analysis by using the observed zone axes to mathematically predict all possible diffraction conditions within the tilt range of the stage. Orientation mapping in this way allows for the extrapolation of imaging conditions which are otherwise easy to miss in the analysis of multiphase materials and allows for self-consistency in their comparison to known crystallography. This method of nanoscale cartography based on the work of Olszta et al. in^50,51^ has been used to great effect in^14^ for the intuitive realization of crystallography inside a transmission electron microscope.

#### APT

APT has been applied in this study to investigate the phase boundary plane with a greater sensitivity to elemental composition than is possible in STEM analysis. Multiple boundary regions were selected and prepared through a site specific liftout process utilizing an FEI Quanta 600 focused ion beam (FIB). APT needles were prepared to present an IPB plane near the tip and have been subjected to a final thinning and cleaning step with 5 keV Ga ions to reduce incident damage at higher ion beam acceleration voltages. It is noted that the dissimilar sputtering and removal rates between the W and γ-phases introduces additional difficulty in the preparation of specimens and only two IPB regions were successfully captured in ten APT needles. For APT analysis a CAMECA local electron atom probe (LEAP) 4000X HR was operated at −233 °C. Data was acquired with a 355 nm laser with a pulse rate of 100 kHz and a detection rate of 0.3% (0.003 detected ions/pulse). Data reconstruction and interpretation was accomplished using the Integrated Visualization and Analysis Software (IVAS) version 3.12.

## Supplementary Information

Supplementary Information.

## Data Availability

The datasets generated during and/or analyzed during the current study are available from the corresponding author on reasonable request.
