# Supplementary material for: Investigation of interfacial strength in nacre-mimicking tungsten heavy alloys for nuclear fusion applications
Source: Sci Rep. 2023 Jan 11;13:575. doi: 10.1038/s41598-022-26574-4 (PMC9834390; doi:10.1038/s41598-022-26574-4)
Supplement: Supplementary file 1 — Supplementary Information. [file 41598_2022_26574_MOESM1_ESM.docx]

***Supplementary Material***

Fitting EDS and APT spectra:

In order to more accurately measure the width of the chemically diffusive boundary as well as to compute the composition of each phase from the bulk, the raw data spectra have been fitted with a sigmoidal function. The equation used to fit this data is of the form:

$y\left( x \right)= \left( \chi_{\gamma}-\chi_{W} \right)\left[ 1-e^{-kx^{n}} \right]+\chi_{W}$ Eq. 1

Where ‘$y$’ is the atomic percentage of the element in question (W, Ni, or Fe) at position ‘$x$’, ‘$\chi_{W}$’ and ‘$\chi_{\gamma}$’ are the atomic composition of the element in the bulk of the W and γ-phases respectively, and ‘$k$’ and ‘$n$’ are constants.

To optimize the functional fit to the acquired data, the difference between the acquired elemental composition and computed composition was taken and squared. This sum of squared differences was computed and minimized through the use of the Solver Add-in for Microsoft Excel. The constants $\chi_{W}$, $\chi_{\gamma}$, $k$, and $n$ were subsequently varied to optimize the fit. The values of $\chi_{W}$ and $\chi_{\gamma}$ correspond to a functionally fitted composition of each element in the bulk W and γ phases respectively and have been displayed in ***Table 1*** of the main text. This solving method has been adapted from two sigmoidal curve fitting protocols by Sparks and Ohgane in Supplementary References [1] and [2] respectively.

To find the maximum slope for the calculation of interphase boundary width by the method detailed by Ardell in [main text reference 30], the derivative of Eq. 1 was taken, providing:

$\frac{dy}{dx}= knx^{n-1}\left( \chi_{\gamma}-\chi_{W} \right)e^{-kx^{n}}$ Eq. 2

The maximum of this equation was determined graphically and applied in the equation from [26] to compute the interfacial width, δ, through the equation:

$\delta= \left| \frac{\chi_{\gamma}-\chi_{W}}{{(\frac{dy}{dx})}_{max}} \right|$ Eq. 3

This method is shown graphically in ***Figures 5*** and ***6*** in the main text, and was applied for each element (W, Ni, and Fe) for each of the four acquired STEM-EDS profiles as well as for each of the four APT reconstructions, providing the $\chi_{W}$, $\chi_{\gamma}$, and $\delta$ values displayed in ***Table 1*** of the main text.


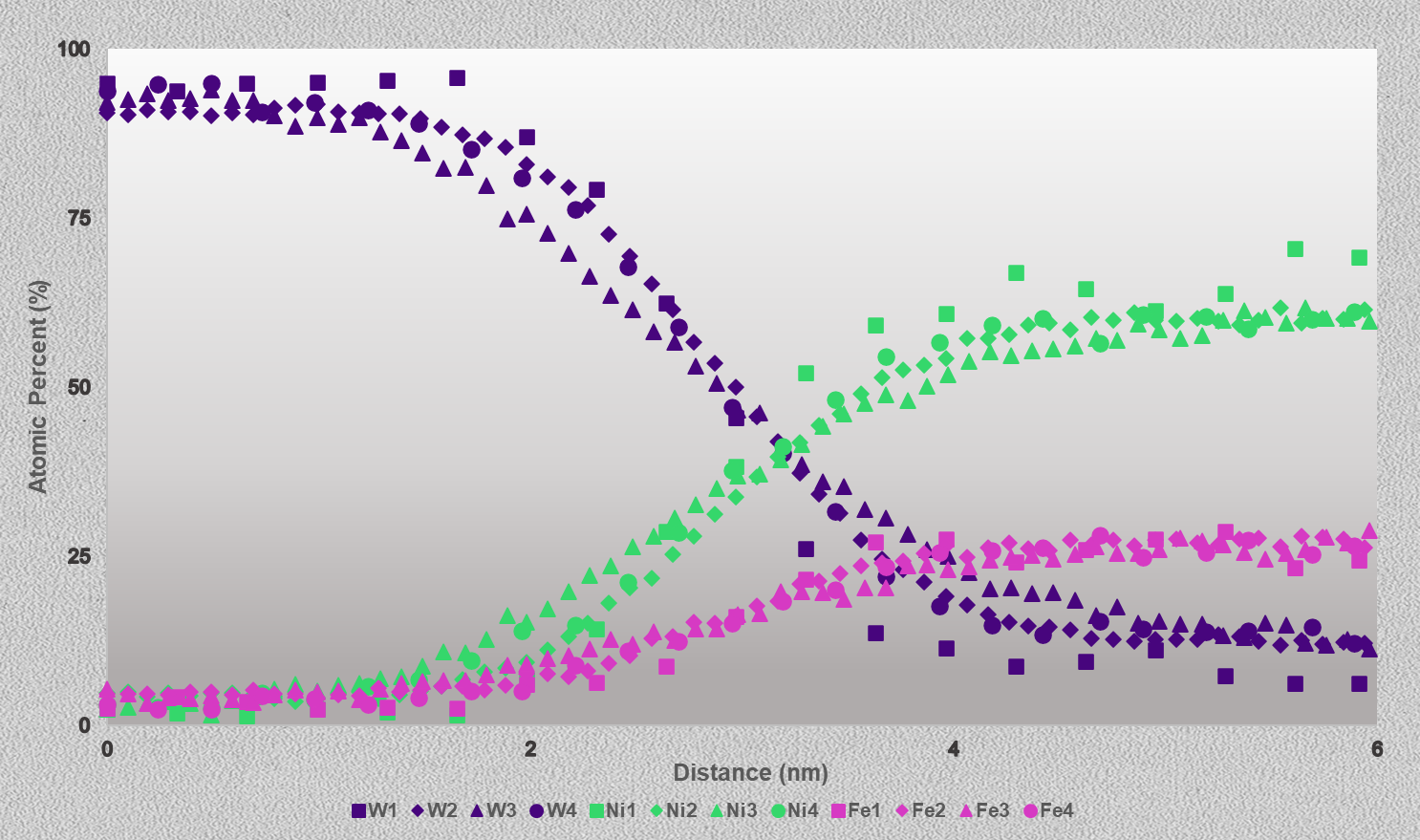


Figure S1: Composite of STEM-EDS line profiles acquired across four different interphase boundary planes. Each boundary has been denoted as 1, 2, 3, or 4 and corresponds to the EDS spectra shown in Figures S.2 through S.5 respectively. Each boundary line scan has been normalized along the x-axis to present the apparent inflection point of the compositional curve at the 3nm point.


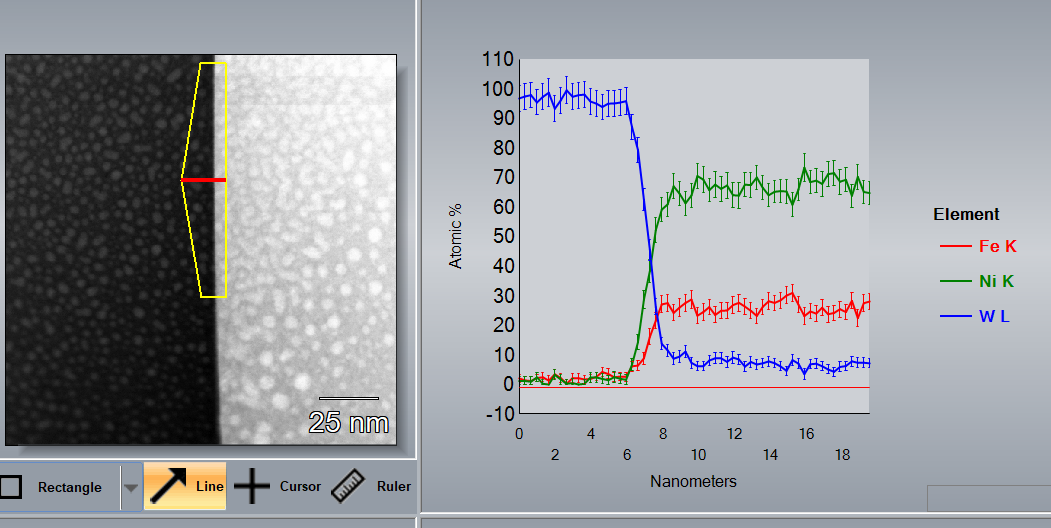


Figure S2: Screen clipping of EDS line profiles acquired across edge-on planar facet at W-γ IPB region. Yellow box with arrow showing directionality corresponds to region averaged to produce line spectra shown denoted as ‘1’ in Figure S1. The composition has been averaged across the direction normal to the arrow to display an averaged composition across the box for greater statistical data collection. Profiles were acquired on JEOL GrandARM at 300kV with a 3C probe and an EDS step size of approximately 0.33nm.


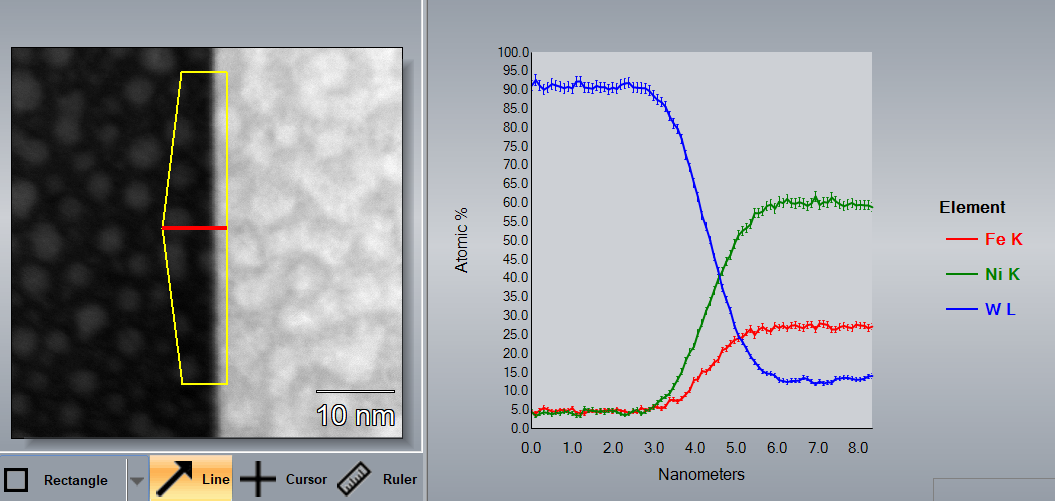


Figure S3: Screen clipping of EDS line profiles acquired across edge-on planar facet at W-γ IPB region. Yellow box with arrow showing directionality corresponds to region averaged to produce line spectra shown denoted as ‘2’ in Figure S1. The composition has been averaged across the direction normal to the arrow to display an averaged composition across the box for greater statistical data collection. Profiles were acquired on JEOL GrandARM at 300kV with a 3C probe and an EDS step size of approximately 0.1nm.


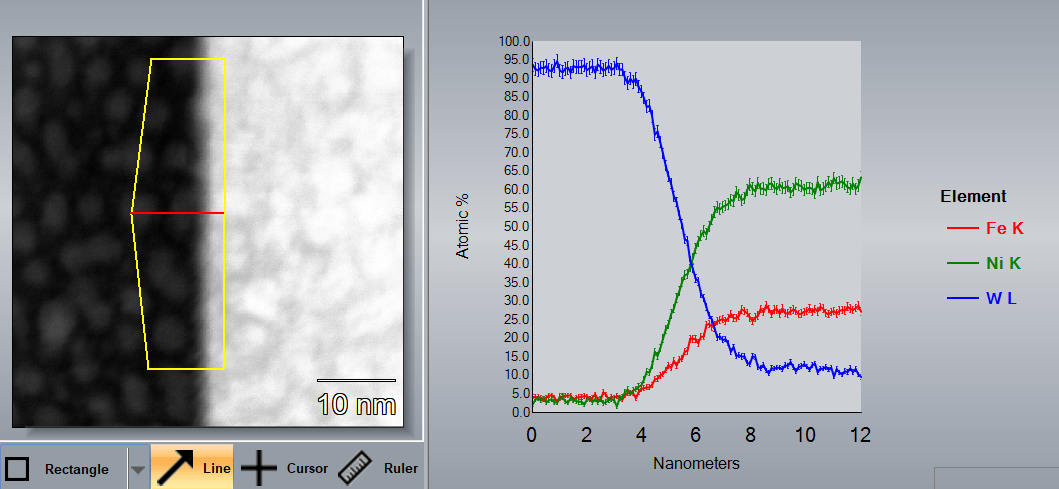


Figure S4: Screen clipping of EDS line profiles acquired across edge-on planar facet at W-γ IPB region. Yellow box with arrow showing directionality corresponds to region averaged to produce line spectra shown denoted as ‘3’ in Figure S1. The composition has been averaged across the direction normal to the arrow to display an averaged composition across the box for greater statistical data collection. Profiles were acquired on JEOL GrandARM at 300kV with a 3C probe and an EDS step size of approximately 0.1nm.


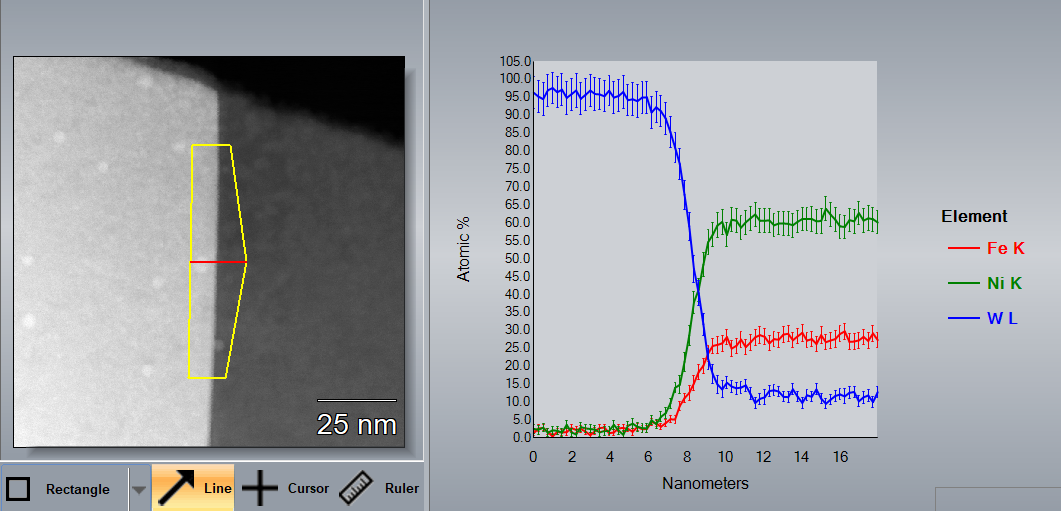


Figure S5: Screen clipping of EDS line profiles acquired across edge-on planar facet at W-γ IPB region. Yellow box with arrow showing directionality corresponds to region averaged to produce line spectra shown denoted as ‘3’ in Figure S1. The composition has been averaged across the direction normal to the arrow to display an averaged composition across the box for greater statistical data collection. Profiles were acquired on JEOL ARM at 200kV with a 5C probe and an EDS step size of approximately 0.25nm.


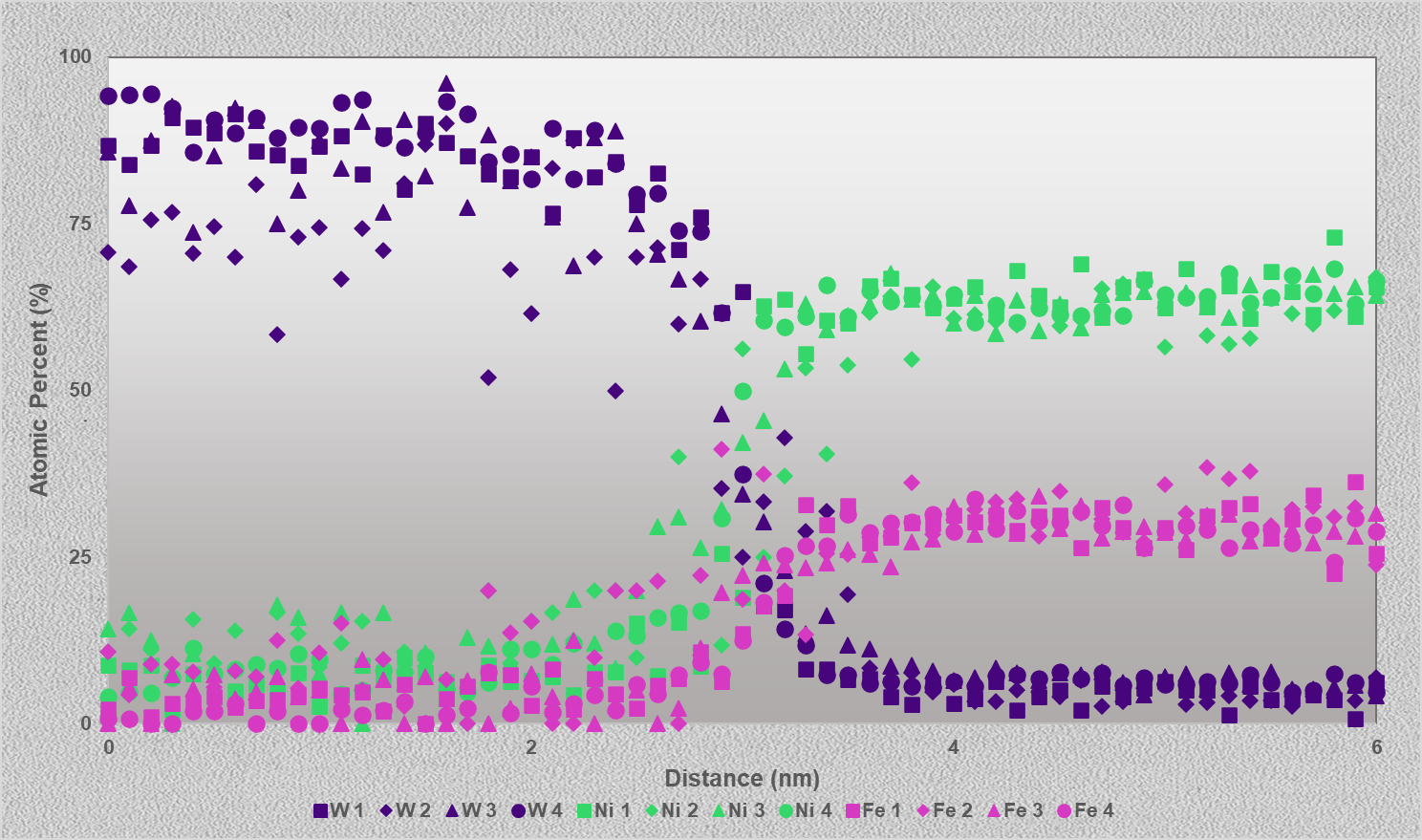


Figure S6: Composite of APT concentration profiles acquired from two regions each from two different APT tips for a total of four specimens. Specimens denoted ‘1’ and ‘2’ are from the APT tip displayed in Figure S7, and have been acquired from the center and edge regions respectively. Specimens denoted ‘3’ and ‘4’ are from the APT tip displayed in Figure S8, and have been acquired from the center and edge regions respectively. Each boundary line scan has been normalized along the x-axis to present the apparent inflection point of the compositional curve at the 3nm point.


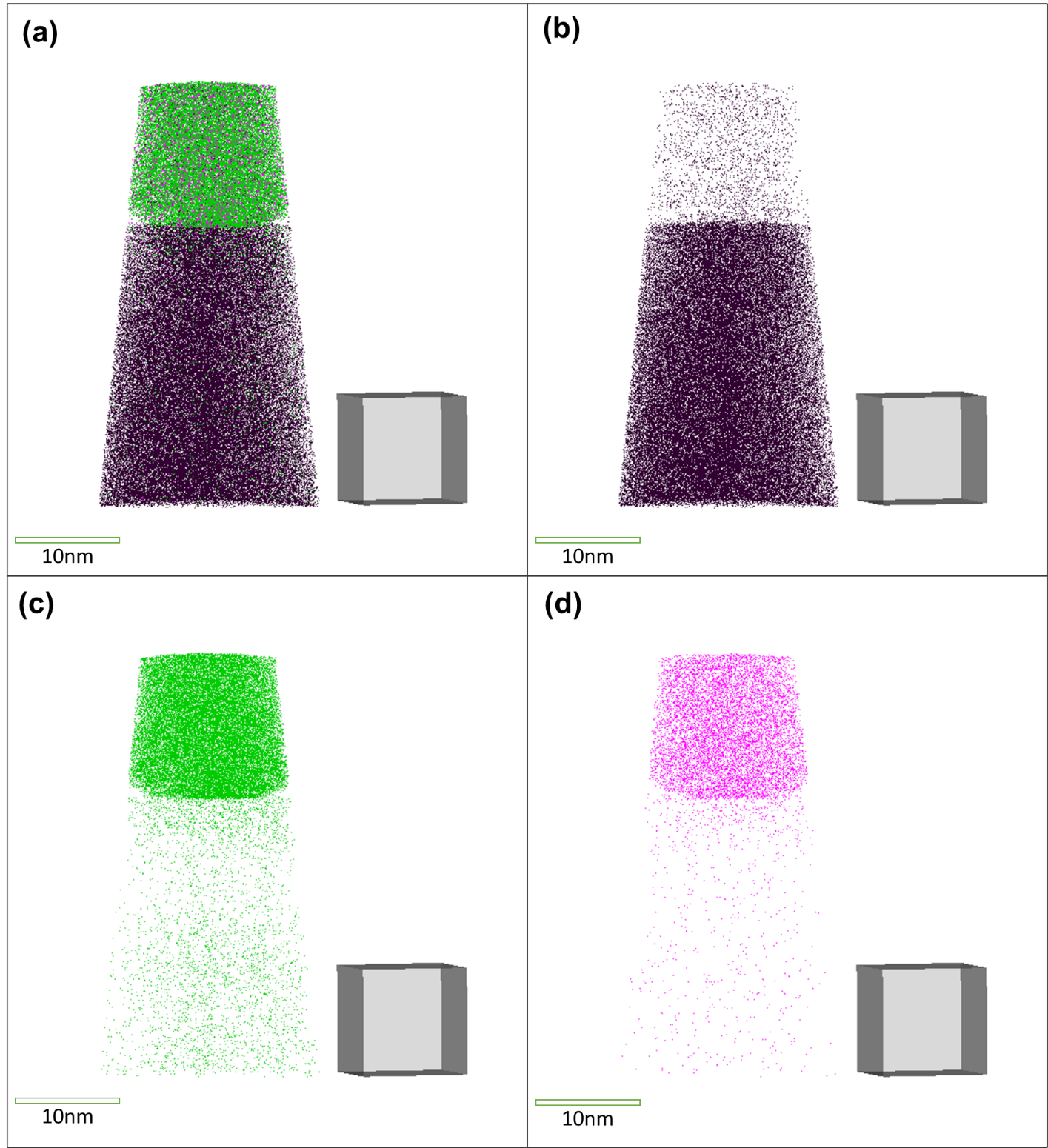


Figure S7: Reconstruction of APT tip from WHA IPB region. (a) ion map composite showing all ions, W in purple, Ni in green, and Fe in pink. (b) ion map showing only W. (c) ion map showing only Ni. (d) ion map showing only Fe. This tip corresponds to specimens ‘1’ and ‘2’ shown in Figure S6.


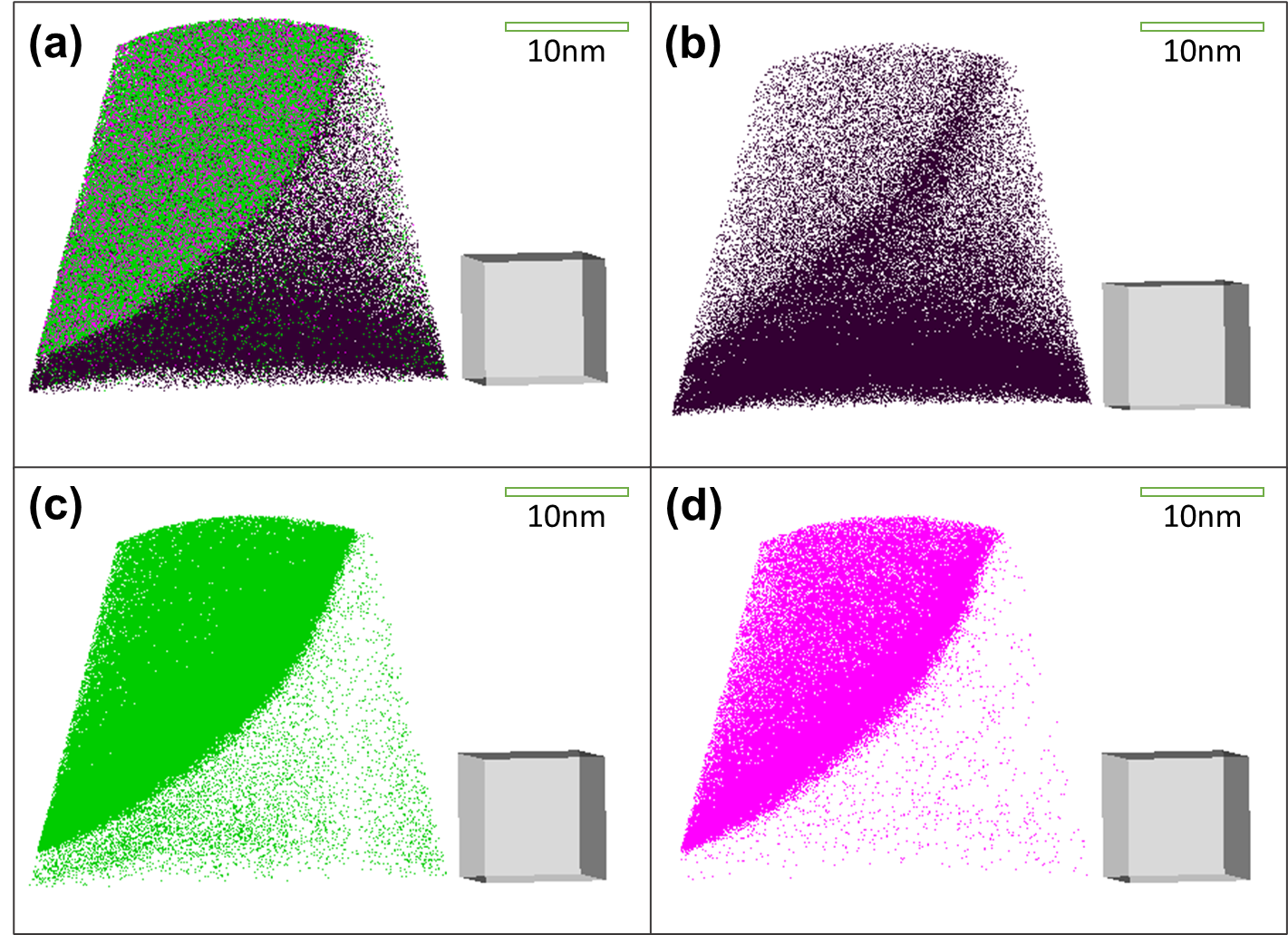


Figure S8: Reconstruction of APT tip from WHA IPB region. (a) ion map composite showing all ions, W in purple, Ni in green, and Fe in pink. (b) ion map showing only W. (c) ion map showing only Ni. (d) ion map showing only Fe. This tip corresponds to specimens ‘3’ and ‘4’ shown in Figure S6.


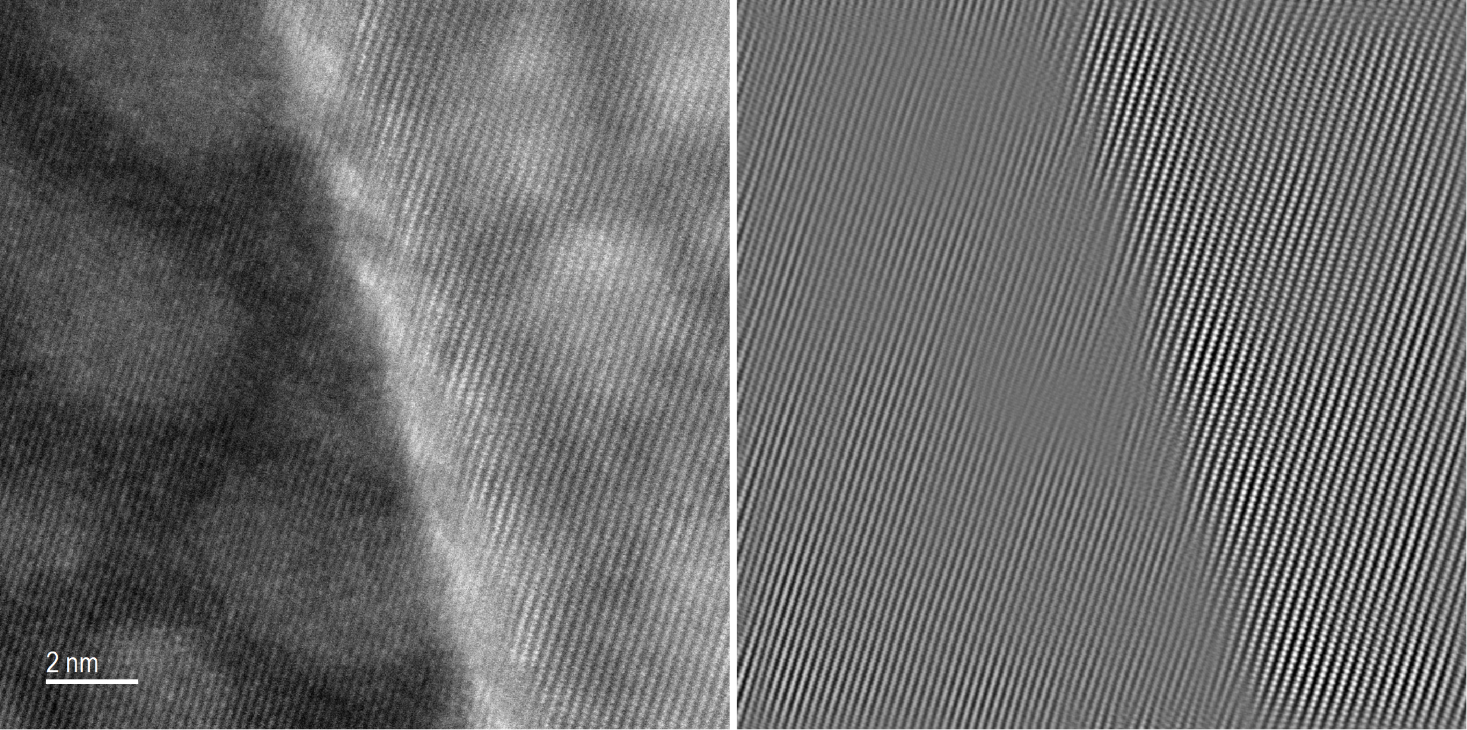


Figure S9: (left) as-acquired STEM image of Facet A shown in the main text. (right) inverse fast Fourier transformed (i-FFT) image to reduce noise for ease of interpretation.


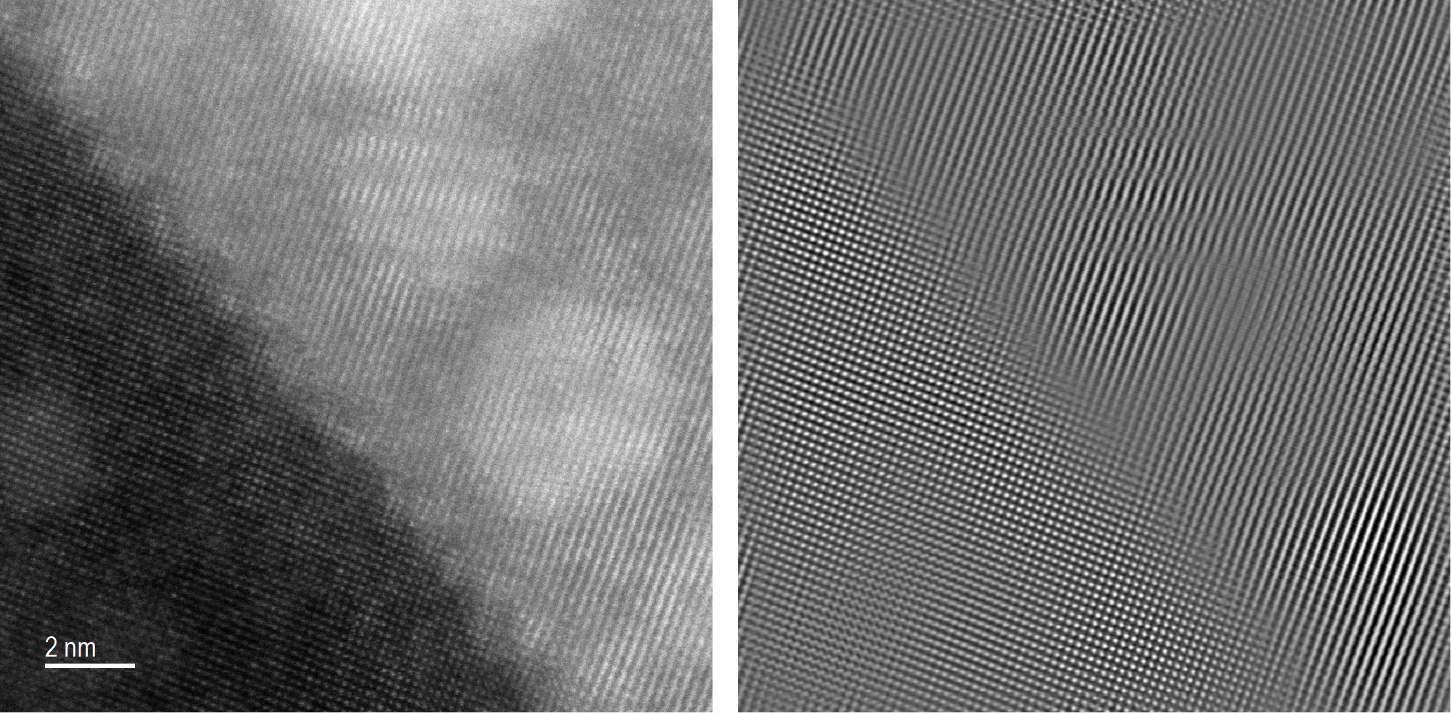


Figure S10: (left) as-acquired STEM image of Facet B shown in the main text. (right) i-FFT image of the same region.


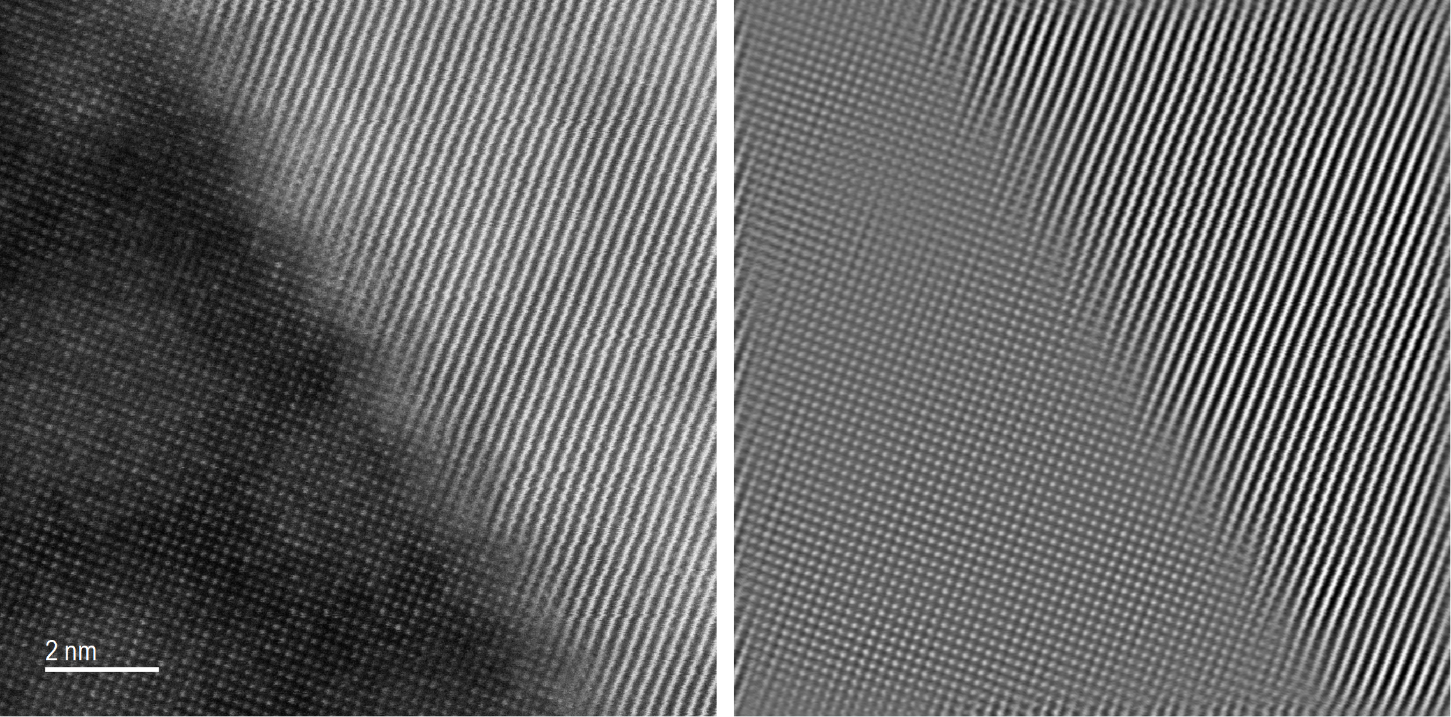


Figure S11: (left) as-acquired STEM image of Facet C shown in the main text. (right) i-FFT image of same region. This is the same image displayed in Figure 4 of the main text with no annotations, showing the stair-stepped ledges at the physical boundary plane in the γ-phase.

Supplementary References:

[1] T. Sparks, "How to fit non-linear equations in excel using solver," YouTube, Nov. 21, 2018, Available: https://www.youtube.com/watch?v=Ewp5CF5ba_w, [Accessed: Aug. 10, 2022].

[2] K. Ohgane, "Sigmoid fitting in Excel (Excel Solver Add-In)," protocols.io, Oct. 14, 2019, Available: https://www.protocols.io/view/sigmoid-fitting-in-excel-excel-solver-add-in-rm7vz8nervx1/v1, [Accessed: Aug. 10, 2022].
